# Biomarkers for Early Detection of Malignant Mesothelioma: Diagnostic and Therapeutic Application

**DOI:** 10.3390/cancers2020523

**Published:** 2010-04-14

**Authors:** Marco Tomasetti, Lory Santarelli

**Affiliations:** Department of Molecular Pathology and Innovative Therapies, Occupational Medicine, Polytechnic University of Marche, via Tronto 10/A Torrette 60020, Ancona, Italy; E-Mail: l.Santarelli@univpm.it

**Keywords:** biomarkers, malignant meosthelioma, asbestos exposure, early diagnosis

## Abstract

Malignant mesothelioma (MM) is a rare and aggressive tumour of the serosal cavities linked to asbestos exposure. Improved detection methods for diagnosing this type of neoplastic disease are essential for an early and reliable diagnosis and treatment. Thus, focus has been placed on finding tumour markers for the non-invasive detection of MM. Recently, some blood biomarkers have been described as potential indicators of early and advanced MM cancers. The identification of tumour biomarkers alone or in combination could greatly facilitate the surveillance procedure for cohorts of subjects exposed to asbestos, a common phenomenon in several areas of western countries.

## 1. Introduction

Malignant mesothelioma (MM) is a rare, highly aggressive neoplasm arising primarily from the surface of serosal cells of the pleural and peritoneal cavities. The incidence of MM is increasing throughout most of the World, and is expected to peak in the US around 2010 [1]; but it is also expected to continue to rise over the next 10 years in Europe [2] as a result of widespread exposure to asbestos in past decades [3]. Asbestos inhalation is the predominant cause of MM, with ~80% of cases of pleural mesothelioma associated with documented asbestos exposure [4]. Although it is well-established that asbestos is the major causative agent in the development of MM, the incidence of cases involving individuals with low levels of asbestos exposure is also increasing.

MM is characterized by a long latency period from the time of asbestos exposure to clinical diagnosis, suggesting that multiple somatic genetic changes may be required for the tumourigenic conversion of mesothelial cells. The evidence of a complex heterogeneity of the structural chromosomal aberrations in MM seems to reflect an intrinsic predisposition of the cells to accumulate genomic damage [5,6]. Difficulty in MM diagnosis and staging, especially of early disease, have thwarted the development of a universally accepted therapeutic approach. MM is notoriously refractory to the different treatment modalities available. Therapeutic options either used alone or combined have been widely tested in the management of MM [7]. Radical surgery with extra-pleural pneumonectomy and adjuvant treatments has become the preferred option in early disease giving benefits in the long term [8]. In cases not amenable to radical surgery, chemotherapy is the first choice over supportive care, whereas platinum-based combination therapy with pemetrexed (Alimta^®^) remains the reference regimen [9].

The management of patients with MM is complicated, first of all because the tumour is notoriously difficult to diagnose. The onset of symptoms is often insidious and non-specific. Therefore, an accurate diagnosis is important for appropriated therapeutic intervention and for proper epidemiological records. Because mesothelioma is fairly well association with asbestos, and exposure is usually in the workplace, it is hypothesized that monitoring a high-risk population might detect patients at an earlier, more treatable stage and result in prolonged survival over the present median 12 months from the start of therapy. Thus, focus has been on finding tumour markers in the blood and other biological fluids that can be used in association with radiography for the non-invasive detection of MM. This review presents recent developments in biomarkers for the early detection of MM.

## 2. Biomarkers

**Figure 1:**
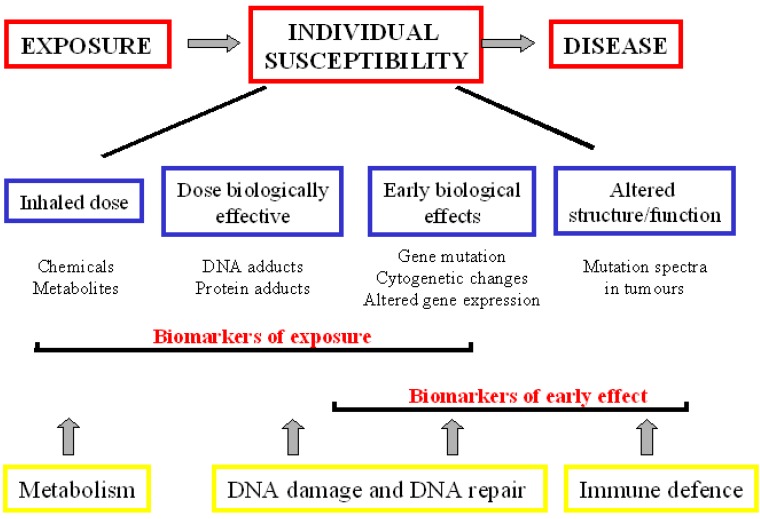
Scheme of biomarkers of exposure, effect and susceptibility in environmental carcinogenesis. The different categories of biomarkers are presented temporally from carcinogen exposure to disease development.

A biomarker is defined as ‘a characteristic that can be objectively measured and evaluated as an indicator of normal and disease processes or pharmacological responses’ [10]. Although the term ‘biomarker’ is relatively new, biomarkers have been used in pre-clinical research and diagnosis for a considerable time. Biomarkers have been widely used to predict, detect and monitor cancer diseases. Human carcinogenesis is the outcome of a complex series of interactions between exogenous (environmental or occupational) factors and endogenous processes, modulated by genetic makeup. Chronic exposure to exogenous mutagenic agents can determine long-term health consequences including cancer development. Therefore, interest has focused on the identification of biomarkers that can be used to monitor an exposed population to improve the prediction of cancer risk (biomarkers of exposure and biomarkers of effect). Other major objectives include the use of biomarker information to clarify the mechanism for disease induction and to provide warming signals that can be used for intervention, diagnosis and therapeutic protocols (functional and diagnostic biomarkers). Different categories of biomarkers are presented temporally from carcinogen exposure to disease development (Figure 1). 

### 2.1. Biomarkers of Exposure

#### DNA Adducts

**Figure 2:**
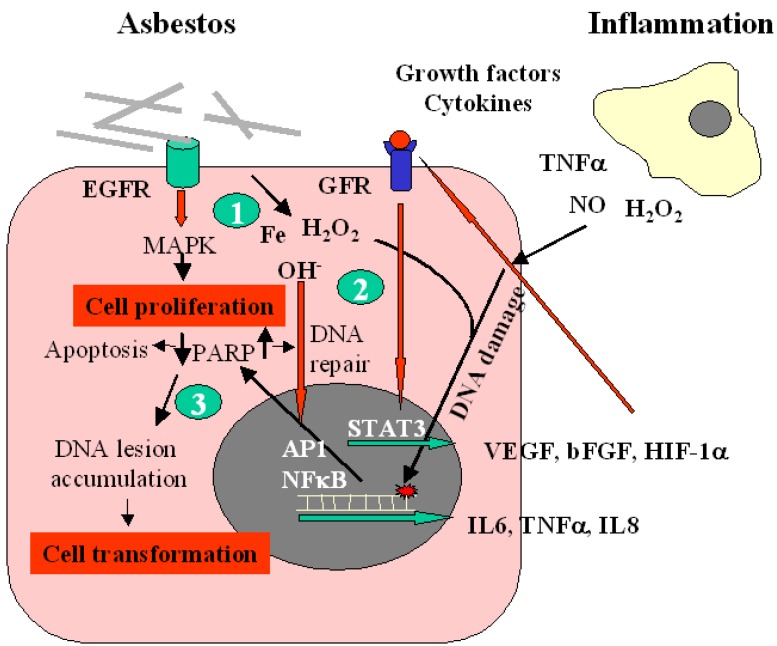
Scheme illustrating the pathogenesis of malignant mesothelioma. Epidermal growth factor receptor (EGFR) is an initial target of asbestos fibers leading to MAPK activation and induction of proliferation (**1**). Reactive oxygen species (ROS) generated directly from asbestos and indirectly from inflammation induce activation of transcriptional factors (AP1, NF-κB) contributing to the regulation of inflammatory cytokines, which in turn interact with their receptors by stimulating the production of growth factors, such as VEGF (**2**). Free radicals generated during inflammation cause DNA damage, including point mutations in cancer-related genes, and modifications in cellular proteins that are involved in DNA repair, apoptosis. Mutagenic DNA lesions that are not repaired accumulate in the genomic DNA of cells thus leading to their transformation (**3**).

Inhaled asbestos fibers with certain physico-chemical properties are known to induce mesothelioma in humans. The genotoxic effects of asbestos may arise due to a number of mechanisms, of which the formation of reactive oxygen or nitrogen species (ROS/RNS) is thought to be particularly important. Following inhalation of asbestos fibers ROS and RNS can be generated in the lung both via Fenton-type reactions catalyzed by iron present on the fibre surface, and via the chronic inflammation induced as a result of prolonged phagocytotic activity of macrophages against the bio-persistent fibers. Asbestos fibers may produce a variety of lesions in cellular DNA, such as single-double strand breaks (SSBs-DSBs), intra-interstrand cross-linking, and base damage [11]. The compound 8-hydroxy-2’-deoxyguanosine (8OHdG), a major product of such oxidative damage [12], causes G→T and A→C transversions [11]. These substitutions have been reported as the sites of spontaneous oncogene expression and may be largely responsible for the onset of carcinogenesis and cell proliferation, ultimately leading to cancer manifestation [12]. The mechanisms involved in the pathogenesis of MM are summarised in Figure 2. 

8OHdG is one of the predominant forms of free radical-induced oxidative lesions, and has therefore been widely used as a biomarker for oxidative stress and carcinogenesis [14,15]. The biomarker 8OHdG is a pivotal marker for measuring the effect of exogenous and endogenous oxidative damage to DNA and as factor of initiation and promotion of carcinogenesis. 

Takahashi *et al.* reported elevated levels of 8OHdG in the DNA of peripheral-blood leukocytes of a population occupationally exposed to asbestos and found that 8OHdG content is related to grade of asbestosis and individual cumulative exposure [16]. Conversely, other authors showed that the high steady-state levels of 8OHdG in the circulating leukocyte DNA of asbestos workers was not correlated with possible confounding factors, such as the presence of benign asbestos-associated diseases, the duration of asbestos exposure, the latency period, the fixed cumulative fibrous dust dose (“fiber years”), age, smoking status, acute febrile infections, medicines, aspirin, calcium (Ca^2+^), magnesium (Mg^2+^), and the hormone and vitamin intake. This indicates that previous inhalation of asbestos fibers is the main factor responsible for the difference observed in oxidative DNA damage between asbestos workers and controls [17]. Although 8OHdG is widely used to estimate the DNA damage in humans after exposure to cancer-causing agents, such as tobacco smoke, asbestos fibers, and heavy metals [18], the suitability of measuring 8OHdG as a biomarker of exposure depends on a range of variables that affect the interpretation of the data. One limitation is the method used for 8OHdG detection. The most common methods for quantitative analysis are high performance liquid chromatography (HPLC) with electrochemical detection (ED) [19], gas chromatography-mass spectrometry (GC-MS), and HPLC tandem mass spectrometry [20]. However, all these methods overestimate the amount of 8OHdG lesions due to the artificial oxidation induced during the procedure of isolation and purification of oxidative DNA products. To solve the methodological problems encountered in measuring quantitatively 8OHdG, two methods have been proposed. One involves the detection of the DNA adduct directly in single cells using comet assay (single-cell gel electrophoresis) by including a step in which the nucleoid DNA is incubated with a lesion-specific endonuclease, which increases the number of breaks and the intensity of the comet tail [21,22]. Endonuclease III (which detects oxidised pyrimidines) or formamidopyrimidine DNA glycosylase (FPG, for 8OHdG) can be incorporated to measure specifically oxidative DNA damage. The other method is based on the direct binding of fluorescent probe to DNA adduct 8OHdG [23]. The FITC conjugated primary antibody bind to 8OHdG in damaged cells and fluorescence is monitored using flow cytometry. Either comet assay or flow cytometry analysis can be used to detect the mutagenic lesion 8OHdG in the nucleoid DNA of cells without introducing further artificial oxidation. 

Recently, using flow cytometry detection, 8OHdG levels have been analysed in the peripheral blood cells of asbestos-exposed workers and MM patients and compared them with age-matched healthy controls [24,25]. Human exposure to asbestos fibers was found to increase significantly the steady-state content of 8OHdG in the lymphocyte DNA of asbestos-exposed workers. Multiple regression analysis revealed that age, smoking status, fibrotic changes and pleural plaques were not important factors in influencing 8OHdG levels. To evaluate whether the 8OHdG content is useful in predicting MM in asbestos-exposed subjects, the receiver operating characteristic (ROC) curves were assessed. Biomarker 8OHdG significantly discriminated the asbestos-exposed population from the age-matched controls but not from MM patients. It is noteworthy that the 8OHdG levels were not evaluated in target (mesothelial) cells but in the surrogate cells (lymphocytes). Thus, the analysis of 8OHdG provides information only about the systemic status that could be affected by the steady-state of mature, newly differentiated and dying lymphocytes, DNA repair, cell division and turnover [15]. The levels of 8OHdG found in lymphocytes depend not only on the life span of the cells but also on the recovery of these adducts and individual blood count variability. The value of 8OHdG levels for predicting cancer on an individual basis is therefore questionable. However, different studies support the notion that the biomarker 8OHdG detects oxidative DNA damage in humans caused by exposure to asbestos fibers, which are involved in the aetiology of MM, but they cannot be used to discriminate between asbestos-exposed individuals with and without MM.

### 2.2. Biomarkers of Early Effect

The main determinant of MM is asbestos exposure. Nevertheless, the interaction between environmental factors and genetic susceptibility might play a critical role in the aetiology of this neoplasm [26]. Heritable differences in host resistance to genetic changes may be identified at different phases of the carcinogenic process, *i.e.*, DNA repair capacity, chromosome stability, cytogenetic changes, modified gene expression, mutation spectra in tumours or pre-cancerous cells.

#### 2.2.1. DNA Repair Ability

DNA repair mechanisms play a key role in limiting the extent of DNA damage and the accumulation of damaged DNA bases. *In vitro* repair of 8OHdG [21] was applied as biomarker assay to investigate the DNA repair ability in workers from factories producing asbestos and man-made fibers [27]. Sixty one asbestos-exposed workers were compared with 21 unexposed factory workers. The authors did not find any differences in repair rates between asbestos-exposed workers and unexposed factory subjects, although the DNA repair activity in exposed female workers showed a lower repair rate than that found in female controls. The study was extended to workers at a stone wool factory where asbestos exposure was found not to affect DNA repair ability [28]. In a our study, the DNA repair ability, evaluated as the persistence of DNA damage over time, was evaluated in the peripheral blood lymphocytes of 42 asbestos-exposed subjects and 25 MM patients and compared with 30 age-matched subjects [unpublished data]. No difference in DNA repair rate was observed between asbestos-exposed subjects and unexposed controls. However, a significant delay in DNA repair was found in MM patients. The relation between DNA repair and cancer risk has been evaluated in some tumours. Using the comet assay, Leprat *et al*. [29] monitored the repair of radiation-induced DNA breaks in lymphocytes from patients with thyroid cancer and found that it was lower than in lymphocytes from healthy controls. Strand break rejoining after the bleomycin treatment of lymphocytes from breast cancer patients [30] or lung cancer patients [31] was delayed when compared with disease-free controls. 

Although environmental exposure and cancer diseases alter the individual DNA repair ability, other variables such as age, sex, lifestyle and nutrition may affect DNA repair. Thus the lack of sensitive, specific, reliable, robust and validated methods make DNA repair assay a questionable biomarker.

#### 2.2.2. Cytogenetic Assay

Cytogenetic damage, measured as chromosomal aberrations in peripheral blood lymphocytes, is a reliable biomarker for human cancer risk independently of exposure to carcinogens [32,33]. Recent evidence suggests the usefulness of a micronucleus test as a screening test for carriers of specific mutations in evaluating cancer susceptibility [34]. A significantly higher rate of micronuclei was found in MM patients and the asbestos exposure was not associated with the high rate of micronuclei [35]. Because about 20% of MM cases occur in subjects without asbestos exposure [36] and only a small percentage of exposed individuals develop the disease, other factors may play a role in mesothelioma development. Therefore, this supports the role of individual susceptibility in determining the risk of MM. Alternatively, the disease itself may somehow play a role in determining the high rate of micronuclei in lymphocytes of MM patients and the lack of a relationship between micronuclei rate and disease progression strengthen the hypothesis that the high rate of micronuclei is a predisposing factor.

### 2.3. Biomarker for Diagnosis

An ideal biomarker for MM should identify patients with MM and differentiate them from patients with other malignancies and subjects at a high risk of developing the disease (asbestos-exposed subjects). The biomarker should be measurable in biological samples collected using non- or minimally invasive tests such as the sampling of blood or pleural fluid. Finally, it should have an acceptable cost. To date, several biomarkers have been proposed for the early detection of MM and their performance (sensitivity and specificity) evaluated as the ability to discriminate patients with MM from subjects without the neoplasm. ROC curves have been used to analyse the diagnostic values of markers individually or in combination.

#### 2.3.1. Conventional Biomarkers

A number of tumour markers in serum and pleural fluid have been evaluated to distinguish malignant effusions from benign ones. Among these parameters cytokeratin fragment (CYFRA 21-1), carcinoembryonic antigen (CEA), carbohydrate antigen 15-3 (CA 15-3), carbohydrate antigen 15-9 (CA 15-9), tissue polypeptid antigen (TPA), and hyaluronic acid (HA) have been found to be of diagnostic significance [37,38,39]. 

CYFRA 21-1, a soluble fragment of cytokeratin subunit 19, showed a significant clinical value in the diagnosis of MM [38,40]. It was reported that mesothelioma cells expressed very high levels of CYFRA 21-1 which were associated with high levels of TPA [41] and low CEA levels [42]. Both CA 15-5 and CA 19-9 appeared to be higher in patients with malignant pleural effusion than in those with benign effusions [37,40,43,44]. However, no significant difference was found in the serum levels of CA 15-5 and CA 19-9 between malignant pleural mesothelioma (MPM) and lung cancer patients [37]. Higher levels of HA were found in MM patients compared with patients with other cancers. However, pleural HA levels could not distinguish MM from benign effusion [45,46]. Recently, the diagnostic value of HA was compared with mesothelin, a potential biomarker for MPM. In pleural fluid, both markers had similar diagnostic values. However, serum HA showed very poor sensitivity and its specificity was found to be significant in diagnosing MPM [47].

In summary, standard markers such as hyaluronic acid, various cytokeratin fragments (CYFRA 21.1, TPA) and other cancer antigens (CA 15.3, CA 125 or CA 19.9 or CEA) are not sensitive or specific enough and cannot be used in clinic practice. More recently new molecules, such as osteopontin and soluble mesothelin have been proposed for diagnostic purposes.

#### 2.3.2. Osteopontin

Osteopontin (OPN) has been described as a promising biomarker for the early detection of MM [48,49]. OPN is a glycoprotein which mediates cell-matrix interactions and cell signalling by binding with integrin and CD44 receptors [50] and is regulated by proteins in cell-signalling pathways that are associated with asbestos-induced carcinogenesis. 

Serum OPN levels were first measured in 69 asbestos-exposed subjects, 45 subjects without asbestos exposure and 76 patients with MM [48]. There were no significant differences in serum OPN levels between age-matched subjects with exposure to asbestos and subjects without asbestos exposure. Serum OPN levels were significantly higher in the group with MM than in the group with exposure to asbestos. With a sensitivity of 77.6% and a specificity of 85.5% at a cut-off value of 48.3 ng/mL ROC analysis revealed that OPN levels discriminate subjects with exposure to asbestos that do not have early MM from those with exposure to asbestos who have early MM. 

More recently, a cross-sectional study evaluated serum OPN levels in an asbestos-exposed population (525 male subjects) to test whether non-malignant asbestos-related disorders could influence OPN levels. There was a significant difference in serum levels of OPN in healthy individuals exposed to asbestos (n = 217) compared with the group of all subjects with asbestos-related disorders (n = 288). Thus, suggesting that OPN, although reported to be useful for detecting MM in asbestos-exposed individuals, may be influenced by non-malignant processes [51]. Another study comparing serum OPN levels from 96 patients with MM and 112 healthy asbestos-exposed subjects showed that serum OPN had a good ability to distinguish between MM patients and asbestos-exposed subjects [52] (Table 1). However, OPN was unable to distinguish MM and pleural metastases carcinoma or benign pleural lesions associated with asbestos exposure [52]. This discrepancy in the evaluation of serum OPN might be because the protein may be cleaved by thrombin during the coagulation process and the results may not reflect the true levels in the blood [52,53,54]. Therefore, serum OPN levels were compared with plasma OPN evaluated in a group of 24 surgically-staged MM patients, in a group of 31 subjects with non-malignant pulmonary diseases and 37 healthy controls [55]. There was no correspondence between serum and plasma OPN measurements (R = −0.1, p = 0.69). Plasma OPN levels did not discriminate between chronic inflammatory and malignant lung diseases and staining intensity in MM specimens did not correlate with OPN plasma levels [55].

**Table 1:** Ability of osteopontin, soluble mesothelin and megakaryocyte potentiating factor to distinguish healthy asbestos-exposed subjects from malignant mesothelioma patients.

Studies	N°	Sample	Biomarker cut-off (ng/mL)	Sensitivity %	Specificity %	AUC
**OPN**						
Pass *et al.* [45]	193	serum	43.3	77.6	85.5	0.89 (0.83–0.93)
Paleari *et al.* [55]	94	plasma	60.8	40.0	100.0	0.60 (0.47–0.72)
Grigoriu *et al.* [52]	208	serum	68.0	95.0	50.0	0.74 (0.68–0.79)
Creany *et al.* [74]	107	serum	18.0	47.0	95.0	0.76 (0.67–0.85)
**SMRPs**						
Grigoriu *et al.* [52]	208	serum	1.7	40.0	100.0	0.74 (0.68–0.80)
Scherpereel *et al.* [61]	137	serum	1.1	71.7	69.8	0.79 (0.73–0.85)
Rodriguez *et al.* [65]	362	serum	1.1	24.0	97.2	0.75 (0.68–0.83)
Amati *et al.* [24]	170	plasma	1.0	90.0	78.0	0.93 (0.88–0.97)
Iwahori *et al.* [66]	121	serum	93.5	59.3	86.2	0.71
Beyer *et al.* [67]	497	serum	1.0	68.2	77.0	–
Cristaudo *et al.* [68]	369	serum	1.0	68.2	80.5	0.77 (0.71–0.83)
Creany *et al.* [74]	107	serum	1.6	73.0	95.0	0.92 (0.87–0.97)
Hollevoet *et al.* [75]	507	serum	2.0	–	64.0	0.87
Creaney *et al.* [77]	233	serum	1.4	–	67.0	0.77
**MPF**						
Iwahori *et al.* [65]	121	serum	19.1	74.1	90.4	0.88
Creany *et al.* [74]	107	serum	1.0	34.0	95.0	0.61 (0.51–0.72)
Hollevoet *et al.* [75]	507	serum	12.4	–	68.0	0.85

Osteopontin OPN, soluble mesothelin-related peptides SMRPs, megakaryocyte potentiating factor MPF, area under curve AUC.

In addition, OPN was found to be over-expressed in colorectal, breast, prostate and lung cancer [56], gastric [57], ovarian [58] cancer and melanoma [59] and the high levels correlate with tumour invasion, progression and metastases. This lack of specificity limit the clinical use of OPN as a diagnostic biomarker for MM.

#### 2.3.3. Soluble Mesothelin-Related Peptides

Mesothelin has been suggested as a promising biomarker for MM [60,61]. Mesothelin is a 71-kDa precursor protein, which undergoes physiological cleavage by a furin-like protease, resulting in two main proteins. One is the 31-kDa NH2-terminal megakaryocyte potentiation factor, which is normally secreted into the blood. The COOH-terminal product of the cleavage, a 40-kDa glycosylated phosphatidylinositol-linked glycoprotein, remains bound to the cell membrane and provides epitopes for immunohistochemistry. It was hypothesized that after further processing by cleavage, the cell surface protein releases soluble mesothelin-related peptides (SMRPs), which are the principal mesothelin family proteins tested for MM diagnosis. 

Mesothelin is constitutively expressed at low levels in mesothelial cells. High levels of SMRP have been found to be associated with MM [60,61], ovarian [62] and pancreatic cancer [63]. Several authors agree with the notion that SMRPs present a useful diagnostic marker for MM. The ROC analysis revealed that the SMRP levels can discriminate MM patients from both the asbestos-exposed and the asbestos-unexposed subjects showing a sensitivity of 60–90% and specificity of 80–85% [24,61,64,65,66] (Table 1). Recently, MM patients were compared with subjects with benign pleural lesions associated with asbestos exposure, and their SMRP levels significantly differentiated the patients with MM from those with benign pleural diseases, with a sensitivity of 80% and specificity of 83% [61,65]. Subjects exposed to asbestos had higher SMRP concentrations than normal control subjects regardless of the presence of pleural disease [24,65]. In addition, serum SMRP levels were higher in patients with MM than in patients with pleural metastases of various carcinomas [61,64,67], or lung cancer [68]. An SMRP level of 1 nmol/L was chosen as the best cut-off to distinguish MM patients from controls (with and without asbestos exposure) [24]. However, it does not discriminate asbestos-exposed individuals from age-matched controls. Thus, the levels of SMRPs in the blood can be proposed as a biomarker suitable for diagnosis of existing MM but not to predict the disease. Beyond its diagnostic applications, SMRP has been suggested as a screening tool able to identify subjects at high-risk of developing MM [69,70]. SMRP levels were measured in the serum of 40 subjects who had a past exposure to asbestos [60]. High levels of serum SMRP were found in seven subjects, of whom three developed MM and one developed lung cancer within five years. Conversely none of the 33 subjects with normal SMRP levels developed the disease after eight years of follow-up. Recently, a large-scale prospective study examined the clinical utility of measuring SMRPs in asbestos-exposed individuals [69]. The study evaluated SMRPs as a potential screening tool for workers in a high-risk population with occupational exposure to asbestos. The results indicated that SMRP is unlikely to prove useful for screening, and is less useful than in diagnosing MM in symptomatic patients. Additional prospective studies performed in a population at high-risk of MM are needed to elucidate the utility of SMRPs as a screening tool.

A meta-analysis study was carried out to evaluate the sensitivity, specificity and measures of accuracy of serum SMRPs in the diagnosis of MM [71]. Summary ROC curves were used to summarize overall test performance. A size sample of 717 patients with MM and 2851 without MM was analyzed. The SRMP levels significantly discriminate the two groups with a sensitivity of 64% (range 41–91%) and specificity of 89% (range 73–100%). The diagnostic accuracy of SMRP determination for MM seems to be similar to that of conventional tests such as cytological examination—high specificity and low sensitivity.

Megakaryocyte potentiating factor (MPF) originates from the same precursor protein of mesothelin; it is potentially more sensitive, yet lacks validation. MPF can be measured by ELISA. Serum levels were to be found higher in MM than healthy subjects, subjects with benign asbestos-related diseases and lung cancer patients [72,73] (Table 1). ROC curves showed an under curve area (AUC) of 0.85 in differentiating MM from controls. The diagnostic performance of MPF as an MM biomarker was compared with the performance of OPN and SMRPs [74,75]. The biomarkers could distinguish patients with MM from healthy controls, whereas MPF and OPN were unable to differentiate patients with MM from patients with other malignancies. A total of 507 participants were enrolled in six cohorts: healthy controls (n = 101), healthy asbestos-exposed individuals (n = 89), patients with benign asbestos-related disease (n = 123), benign respiratory disease (n = 46), lung cancer (n = 63) and MM (n = 85) and analysed for SMRPs and MPF levels. The similar AUC values of SMRPs and MPF, together with the limited difference in sensitivity, showed that both serum biomarkers had an equivalent diagnostic performance [76]. 

#### 2.3.4. Biomarker Combination

Due to the limitations of single biomarkers in terms of sensitivity and specificity much effort has been focused on the use of a biomarker combination that can distinguish between asymptomatic asbestos-exposed subjects and early-stage MM patients. A biomarker panel such as CEA, CA 15-3, CA-125, CYFRA 21-1 in various combinations increase the diagnostic accuracy in distinguishing malignant pleural effusion from benign effusion [37,43,44]. However, one study reported contradictory results, showing that biomarker combinations did not perform better than CEA alone [76]. To improve the diagnostic accuracy of markers some of them have been combined with mesothelin. A panel consisting of CYFRA 21-1, CEA and soluble mesothelin was evaluated by ROC analysis to discriminate between MM patients and healthy subjects [40]. The combinations weakly improved the biomarker performance compared to CYFRA 21-1 alone. Likewise, the combination of soluble mesothelin with CA-125 [77] or with MPF and OPN [74] did not improve sensitivity for detecting MM compared to the mesothelin marker alone.

Recently, a combination of biomarkers has been proposed [24]. Levels of the DNA adduct 8OHdG (marker of exposure), factors involved in tumour growth such as Platelet derived growth factor (PDGF), hepatocyte growth factor (HGF), basic fibroblast growth factor (bFGF), vascular endothelial growth factor (VEGF), progression including metalloproteinases (MMP2 and MMP9), tissue inhibitor metalloproteinases (TIMP1 and TIMP2) and SMRPs (a specific biomarker of MM) were assessed in high-risk asbestos-exposed subjects, patients with MM and healthy subjects. Despite lacking specificity, the levels of 8OHdG, HGF, bFGF and VEGF alone distinguished high-risk subjects from healthy persons and MM patients. The combination of SMRPs with 8OHdG and the growth factor VEGF highly increased the sensitivity and specificity to discriminate the high-risk subjects from healthy controls. This panel of biomarkers might be used to stratify the risk of MM in individuals with a history of asbestos exposure [24] (Table 2). 

**Table 2:** Ability of 8OHdG, VEGF, SMRPs and their combination to distinguish age-matched subjects and asbestos-exposed subjects from malignant mesothelioma patients.

Marker	Ctrl *vs.* Exp (AUC)	Exp *vs*. MM (AUC)	Ctrl *vs.* MM (AUC)
8OHdG (AU)	0.775 ± 0.037	0.566 ± 0.110	0.788 ± 0.090
VEGF (ng/mL)	0.714 ± 0.062	0.705 ± 0.086	0.803 ± 0.074
SMRPs (nM)	0.459 ± 0.042	0.927 ± 0.022	0.920 ± 0.030
8OHdG-VEGF-SMRPs	0.925 ± 0.035	-	-

ROC curve analysis performed in 54 control subjects (Ctrl), 94 asbestos-exposed subjects (Exp), and 22 MM patients (MM). An area under ROC curve (AUC) of 1.0 indicates perfect discrimination, whereas and area of 0.5 indicates that the test discriminates no better than chance. 8-hydroxy-2’-deoxyguanosine 8OHdG, vascular endothelial growth factor VEGF, soluble mesothelin-related peptides SMRPs.

#### 2.3.5. Molecular Biomarkers

There is emerging evidence for the role of molecular changes in lung diseases such as lung cancer and MM as responses to environmental exposures. Insight into epigenomics will lead to the development of novel biomarkers and treatment targets in tumour diseases. Deregulation of epigenetic transcriptional control (aberrant promoter DNA hypermethylation and histone acethylation) is a fundamental feature of human malignancies [78]. In lung cancer and mesothelioma, a number of genes involved in carcinogenesis have been demonstrated to be hypermethylated, implicating epigenomic changes in the aetiology of these cancers. Hypermethylated genes have also been associated with lung cancer recurrence, indicating epigenomic regulation of metastasis [79]. The relation between promoter DNA hypermethylation and inflammation has been described in many types of tumours [80]. Asbestos exposure might contribute to MM development through this relationship [81,82]. It is known that asbestos induces continuous inflammation instead of directly transforming human mesothelial cells [83]. 

There is increasing evidence that hypermethylation of CpG islands can be one of the most prevalent molecular markers for human cancers (epigenetic biomarkers). Changes in the status of DNA methylation and chromatin modifications are characteristics of neoplastic cells and the different pattern can be useful in diagnosis and classifying tumours. There are several advantages in using hypermethylated genes as biomarkers. First, the DNA is a highly stable molecule and can be obtained from a wide variety of sources. Secondly, methylation-specific PCR, high-performance liquid chromatography (HPLC), and high-performance capillary electrophoresis (HPCE) are sensitive techniques for methylated gene detection. Elevated amounts of free-circulating nucleic acids were found in cancer patients compared with controls [84] and these free nucleic acids can be used to detect cancer-related molecular alterations. A hypermethylation pattern of serum DNA was reported for MM [85]. The combination of three genes (DAPK, RASSF1A, RARb) significantly correlated with survival of MM patients. 

Multiple epigenetics play an important role in gene expression whose abnormality might be reflected in the alteration of the expression of genes. It is known that epigenetic mechanisms are involved in the regulation of microRNAs (miRNAs), a class of non-coding RNAs [86,87]. miRNA can be targeted by epigenetic modification, as well miRNAs can target regulators of epigenic pathway.

miRNAs were found to regulate post-transcriptionally the expression of target genes and may behave as oncogenes or tumour suppressors. Shortly, after their discovery, miRNAs were found to be associated with cancer [88,89]. Accumulating reports highlight the potential diagnostic utility of miRNAs in cancers [90,91,92]. miRNA expression profile can be used to distinguish normal from malignant tissues, to identify the tissue origin in poorly differentiated tumours or tumours of unknown origin and to distinguish the different subtypes of the same tumour. Earlier reports have show the presence of tissue-specific signatures of miRNA expression in MM. Using miRNA microarray, 723 human and 76 viral miRNAs were analysed in fresh frozen biopsies obtained from 17 MM and compared with normal human pericardium [93]. The results clearly distinguish the tumour profile from the normal tissue profile: twelve miRNAs were highly expressed, whereas nine were down-regulated. 

More recently, Busacca *et al.* [94] evaluated, by microarray profiling, the miRNA expression on mesothelioma cell culture. The significantly deregulated miRNAs were confirmed by quantitative reverse transcription-PCR (qRT-PCR) and subsequently analysed on twenty-four MM specimens, representative of three histotypes (epithelioid, biphasic and sarcomatoid). A panel of deregulated miRNA was found. 

The two studies which were aimed at identifying a tumour-specific miRNA profile reported different results. This suggests that the biological sample and the methodological approach used both affect the data. To identify a specific miRNAs signature of MM better, we used fresh-frozen biopsies of MM and the miRNA expression quantified by qRT-PCR miRNA array. Eighty-eight miRNAs involved in cancer development were assayed in the fresh frozen biopsies of MM and compared with normal mesothelium tissues. After statistical analysis, only eight were significantly under-expressed and their down-regulation was more evident in advanced tumours. Three mainly deregulated miRNAs were then analysed in the larger sample series using formalin-fixed paraffin-embedded (FFPE) sections. By analysing FFPE tissues, two miRNAs were identified as significantly down-regulated in pathological conditions (unpublished data). 

Recently, finding miRNA in the blood has suggested the potential for miRNA-based blood biomarkers in cancer detection [95,96]. It was hypothesised that the levels of specific circulating miRNA species may be used to detect and monitor the pathological development associated with agent-induced tissue injuries. Evaluating miRNA levels in the serum of a population of asbestos-exposed subjects, MM patients and healthy age-matched controls, we observed that low levels of miRNAs were found in the blood of MM patients and high-risk subjects (asbestos-exposed subjects) when compared with controls. miRNAs might be a marker for early diagnosis and prognosis of MM and exposure to asbestos. 

Even though several studies have reported the potential use of miRNAs as biomarkers to be found in the serum, several topics still need to be further refined. First, a new and robust standardization method is required to obtain accurate and reproducible results. Second, large studies reporting the distribution of miRNAs in the serum of a normal population are needed. Finally, a better understanding of the mechanism by which miRNAs are released in the circulation is required. Therefore, a new strategy of utilising miRNA profiling as an adjuvant diagnostic or prognostic tool without compromising the clinical diagnosis has emerged. It is unlikely that miRNA analysis will replace the existing tools for tumour diagnosis and management. A practical view is that they will be added in conjunction with the existing tools.

Epigenetic changes and miRNA deregulation both affect gene expression. Using the genome-wide expression analysis novel genes associated with MM were identified [97,98]. Notable, is the identification of MMP-14, a member of matrix metalloproteinase family, as a diagnostic and prognostic marker [97]. A prognostic predictor was identified using the relative expression of four other genes in a training set of 39 patients with validation in a test set of 52 patients; the test had a 69% accuracy, but was not significant in multivariate analysis [99]. Using microarray-based tumour expression data from 21 patients with MM, a 27-gene classifier was developed by neural network modelling weighted according to survival. The results were validated using hierarchical clustering in a test set of 17 patients with MM with a accuracy of 76% [100]. Gordon *et al.*, developed a gene ratio test to predict the outcome of malignant pleural mesothelioma patients undergoing surgery. They concluded that the gene ratio test for survival of patients with MM has a robust predictive value and technical assay performance [101].

### 2.4. Prognostic Biomarkers

Since the progression of the MM differs among individuals, a number of scoring systems based on assessment of clinicopathological features of patients with the disease have been developed but the search continues for further prognostic indicators. Biomarkers are needed to determine the disease aggressiveness. Validated biomarkers would not only have an impact on clinical practice, but would also be used for the stratification of patients in clinical trials. 

CYFRA 21-1, TPA and HA have been suggested as prognostic factors in retrospective studies [102,103,104]. However, only CYFRA 21-1 was considered as an independent prognostic factor in multivariate analysis. 

Neoangiogenesis may be considered a critical step in the development of MM. PDGF and VEGF are autocrine growth factors in MM and the epidermal growth factor receptor (EGFR) appears highly expressed in this tumor. It was reported that MM patients having serum PDGF levels below or above 49.8 ng/mL had a median survival of 13.1 and 7.9 months, respectively [105]. However, the higher PDGF levels were not significantly associated with shorter survival when adjusted for age, sex, histology and platelet count. Several studies have reported that VEGF plays an important role in angiogenesis in MM tumours [106,107]. However, a question remains as to whether VEGF has a relationship with its prognosis. Serum levels of VEGF were found to be inversely correlated with the survival of MM patients, but the clinic utility of VEGF as prognostic factor was not evaluated [107].

EGFR is one of the ErbB families of receptor tyrosine kinases. These cell membrane receptors play a central role in cell proliferation, differentiation, migration, adhesion and survival [108]. Immunohistochemical expression of EGFR was significantly associated with longer survival, not independent of other prognostic factors [109,110]. The absence of EGFR immunoreactivity also correlated with other well-established predictors of poor prognosis, such as the presence of chest pain, weight loss and poor performance status [110]. Similar conclusions were draw concerning the estrogen receptor-β (ER-β). Immunohistochemical analysis revealed intense nuclear ER-β staining in normal pleura that was reduced in tumour tissues. Multivariate analysis of 78 MM patients with pathologic stage, histologic type, therapy, sex and age at diagnosis indicated that ER-β expression is an independent prognostic factor of better survival [111].

Resistance to apoptosis is one of the main reasons for drug resistance and treatment failure. The expression of several proteins involved in apoptotic pathways were examined by immunohistochemistry. No association with survival was found for Bcl-2, Bcl-x, Mcl-1 and Bax [112]. However, a study that included tumour samples from erionite-induced MM showed that the immunohistochemical expression of Bax was an independent prognostic factor for MM [113]. The same authors also showed that immunohistochemical expression of Fas ligand, a protein involved in the extrinsic apoptotic pathway, was correlated with MM survival [113]. Recently, a study exploring several inhibitors of apoptosis such as IAP-1, IAP-2, livin, survivin and XIAP in MM samples found that only survivin and IAP-1 were associated with shorter survival, while XIAP and livin were associated with longer survival [114]. However, the independence of these biomarkers as prognostic factors was not assessed.

The modification of the extracellular matrix (ECM) is a fundamental step in tumour invasion and the prognostic value of ECM protein expression and activity have been assessed in MM. Among the matrix metalloproteinases that are involved in ECM remodelling, MMP-2 but not MMP-9 was found to be a significant and independent factor for poor prognosis [115]. 

Finally, a prognostic value for MM was found for cyclo-ocygenase (COX) enzyme which plays a central role in arachidonic acid metabolism. High expression of COX-2 in MM specimens was demonstrated to be an independent predictor of poor prognosis [116,117].

### 2.5. Target Biomarkers

Mesothelioma is a malignancy which owes its chemoresistance to an apoptotic defect. Thus the introduction of new biologic drugs could provide the best results for MM treatment. A number of target agents have been tested in MM [118], but none of these have been targeted to molecular alterations specific for MM. 

Tyrosine kinase inhibitors (TKIs) targeting growth factors and angiogenesis inhibitors are among the most promising agents under evaluation in clinical trials. VEGF can be blocked by monoclonal antibodies (bevacizumab), by TKIs (semaxanib, sunitinib, vatalanib, sorafenib) and indirectly by drugs that interfere with the synthesis of growth factors (thalidomide).

The use of a number of angiogenesis inhibitors has been or is being investigated. Pivotal trials with PTK787 and thalidomide have demonstrated little activity [119,120]. Some efficacy has been reportedfor SU5416, an inhibitor of the VEGF receptor (VEGFR) Flk-1, but this was hampered by an excessive risk of thrombosis [121]. Bevacizumab, a recombinant human anti-VEGF monoclonal antibody that blocks the binding of VEGF to its receptors, is under evaluation in a double-blind, placebo-controlled, randomized phase II trial in combination with cisplatin and gemcitabine. Other novel agents under investigation include sorafenib, an inhibitor of VEGFR-2, PDGFR, and the B-Raf tyrosine kinase [122]. No significant effects were reported and no objective responses were registered in 33 patients with MM.

EGFR has been the subject of much interest in the last few years as a target for selective EGFR TKIs and anti-EGFR monoclonal antibodies [123]. EGFR inhibitors have been tested in MM patients although the results have not been conclusive so far. A phase II study of gefitinib administrated as a single agent to 43 patients with MM has recently been reported [124]. Two patients had a radiological response and 21 had a stable disease, although survival rates were not greater compared to the historic CALGB registry data. Another phase II study investigated the effects of erlotinib in 64 MM patients and examined these effects with respect to the expression of EGFR, phospho-EGFR, HER2, phospho-ERK, phospho-AKT and PTEN [125]. 

In a phase I trial, vorinostat, a histone deacetylase inhibitor, produced objective responses in 20% of MM patients, and a phase III double-blind, placebo-controlled trial is under way [126]. Bortezomib, a proteasome inhibitor, has shown activity in pre-clinical models of MM, acting via mechanisms including the inhibition of angiogenesis and NF-κB, the latter having been reported as a crucial cellular effector of chemoresistance in MM [127].

Resistance to apoptosis is a characteristic feature of MM [128]. For example, members of the inhibitor of apoptosis protein (IAP) family are regulated by TNFα in pleural MM. Mesothelioma cells exposed to TNFα were twice as resistant to cisplatin as were unstimulated controls and were found to have a significantly greater fraction of surviving cells at high cisplatin concentrations [129]. Therefore, the most promising therapeutic approaches for MM are likely to be those directed at eradicating cancerous cells by targeting the relevant apoptotic pathways [130,131].

### 2.6. Predictive Biomarkers

Predictive biomarkers are used to predict the outcomes of treatments. Few studies have focused on predictive biomarkers to monitor the response of MM to treatment. Some serum markers might be used as disease monitoring tools to predict the success or failure of MM treatment. Serum mesothelin measured at the time of diagnosis has been shown to be correlated with tumour volume [60] and survival [54]. In addition, some studies [60,132,133] reported that soluble mesothelin levels were associated with disease progression and worse outcome, whereas stable or decreased values suggested a response to treatment. These results support the use of serum mesothelin in monitoring treated patients with MM. However, due to the small number of recruited patients these finding should be interpreted with caution. Further prospective investigations are needed to establish the use of serum mesothelin levels as a predictive marker in patients with MM.

## 3. Conclusions

**Figure 3:**
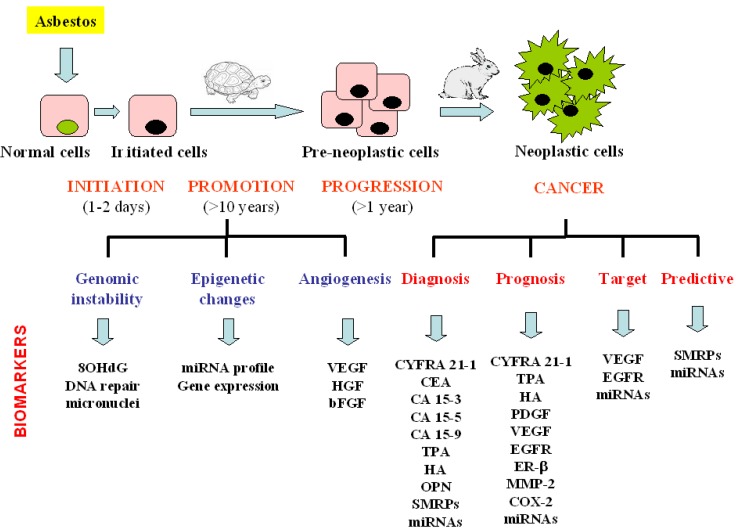
Schematic presentation of biomarkers evaluated from asbestos exposure to malignant mesothelioma development. MM is characterised by a long latency period from the time of exposure to clinical diagnosis. The biomarkers that can be detected at the different phase of the malignant disease development are summarised.

Biomarkers might be helpful in managing three clinical aspects of MM: early diagnosis, prognosis and treatment outcome prediction. The biomarkers that can be detected at the different phase of the malignant disease development are summarised in Figure 3.

Whereas a large number of biomarkers have been assessed in biological fluids and tumor tissue for their prognostic value, none have had a widespread impact on clinical practice.

8-hydroxy-2’-deoxyguanosine (8OHdG), vascular endothelial growth factor (VEGF), hepatocyte growth factor (HGF), basic fibroblast growth factor (bFGF), cytokeratin fragment (CYFRA 21-1), carcinoembryonic antigen (CEA), carbohydrate antigen 15-3 (CA 15-3), carbohydrate antigen 15-5 (CA 15-5), carbohydrate antigen 15-9 (CA 15-9), tissue polypeptid antigen (TPA), hyaluronic acid (HA), osteopontin (OPN), soluble mesothelin-related peptides (SMRPs), megakaryocyte potentiating factor (MPF), platelet derivate growth factor (PDGF), epidermal growth factor receptor (EGFR), matrix metalloproteinases-2 (MMP-2), cyclo-ocygenase-2 (COX-2).

Most recently, serum biomarkers with the potential to discriminate individuals exposed to asbestos without cancer from those with MM have been investigated both at single institutions and with multi-institutional-blinded trials. These markers include OPN, SMRPs, and MPF. However, OPN lacks specificity for mesothelioma, while both SMRPs and MPF lack sensitivity for detecting non-epithelial subtypes. An improved sensitivity to distinguish asbestos-exposed subjects from healthy non-exposed individuals was obtained when non-specific biomarkers of exposure such as 8OHdG and factors involved in tumour growth (VEGF) were combined. Biomarker combination may, in the future, be incorporated into a screening algorithm for high-risk asbestos-exposed individuals to help monitor these cohorts in a non-invasive way and guide the use of computerized tomography. In contrast, data concerning predictive biomarkers are very limited, even though they are very interesting from the perspective of clinicians. Additional prospective studies, in large and independent samples of patients, with rigorous statistical methodology and standardized laboratory techniques are now warranted to validate and define the precise value of diagnostic and prognostic MM biomarkers. Future research should focus on biomarkers that predict the efficacy and toxicity of standard chemotherapy. Translational research should be systematically incorporated into the design of clinical trials assessing new targeted agents in MM.
